# Metacognitive Feelings: A Predictive-Processing Perspective

**DOI:** 10.1177/17456916231221976

**Published:** 2024-01-29

**Authors:** Pablo Fernández Velasco, Slawa Loev

**Affiliations:** 1 Department of Philosophy, University of York; 2 Philosophy of Science and the Study of Religion, Ludwig Maximilian University of Munich

**Keywords:** metacognitive feelings, predictive processing, interoceptive inference, affect, mental action, cognition, prediction, emotion

## Abstract

Metacognitive feelings are affective experiences that concern the subject’s mental processes and capacities. Paradigmatic examples include the feeling of familiarity, the feeling of confidence, or the tip-of-the-tongue experience. In this article, we advance an account of metacognitive feelings based on the predictive-processing framework. The core tenet of predictive processing is that the brain is a hierarchical hypothesis-testing mechanism, predicting sensory input on the basis of prior experience and updating predictions on the basis of the incoming prediction error. According to the proposed account, metacognitive feelings arise out of a process in which visceral changes serve as cues to predict the error dynamics relating to a particular mental process. The expected rate of prediction-error reduction corresponds to the valence at the core of the emerging metacognitive feeling. Metacognitive feelings use prediction dynamics to model the agent’s situation in a way that is both descriptive and directive. Thus, metacognitive feelings are not only an appraisal of ongoing cognitive performance but also a set of action policies. These action policies span predictive trajectories across bodily action, mental action, and interoceptive changes, which together transform the epistemic landscape within which metacognitive feelings unfold.

Through all he said, even through his appalling sentimentality, I was reminded of something—an elusive rhythm, a fragment of lost words, that I heard somewhere a long time ago. For a moment a phrase tried to take shape in my mouth and my lips parted like a dumb man’s, as though there was more struggling upon them than a wisp of startled air. But they made no sound and what I had almost remembered was uncommunicable forever.

—F. Scott Fitzgerald, *The Great Gatsby*

The feeling described in the quote above is an incidence of the tip-of-the-tongue (ToT) experience, which occurs when a person is trying to retrieve an item that is temporarily inaccessible (R. Brown & McNeill, 1966; B. L. Schwartz & Metcalfe, 2011). When most people think about feelings, emotions such as fear, anger, or sadness might come to mind, not necessarily the ToT experience. The latter seems to belong to a particular category that has been the topic of much recent discussion in the cognitive sciences: metacognitive feelings (Arango-Muñoz, 2013; Koriat & Levy-Sadot, 1999), sometimes also referred to as epistemic (de Sousa, 2008; Proust, 2009a), noetic (Dokic, 2012), or simply cognitive (Greifeneder et al., 2011) feelings. Metacognitive feelings have been characterized as “feelings concerning the subject’s own mental capacities and mental processes” (Arango-Muñoz & Michaelian, 2014, p. 97); “feelings that enter into the epistemic processes of inquiry, knowledge and metacognition” (de Sousa, 2008, p. 198); or “feelings about knowing” (Clore, 1992).

A big open question about metacognitive feelings concerns their underlying mechanism. We know that metacognitive feelings correlate with certain process properties, most notably process fluency (Alter & Oppenheimer, 2009; Whittlesea & Williams, 1998, 2001a, 2001b), but there is no encompassing theory of how they emerge. We know that metacognitive feelings influence mental action, but there is no definite notion of the way in which they do so. Predictive processing (PP), a novel theoretical framework that considers the brain a hierarchical prediction machine, provides a promising way to tackle these issues. In this article, we offer an account of metacognitive feelings within the framework of PP in an effort to shed light on how metacognitive feelings emerge and how they guide mental action. We first review previous research on metacognitive feelings. We then introduce PP, as well as existing accounts of emotions within this framework. Finally, we build on this work to propose a PP account of metacognitive feelings.

## Metacognitive Feelings

A key distinction in the psychological literature separates metacognitive feelings from metacognitive judgments (Koriat & Levy-Sadot, 1999). The latter are based on an explicit and deliberate inferential process. In contrast, metacognitive feelings are experiences that emerge out of the implicit and automatic evaluation of cognitive processes.

Early work on metacognitive feelings involved studies of the feeling of knowing (FoK; Hart, 1965) and ToT (R. Brown & McNeill, 1966) experiences. This line of work, however, only properly took off when Flavell (1979) outlined a framework in which he identified metacognitive experiences as a distinctive facet of metacognition. Further theoretical developments arrived with Nelson and Narens’s (1994) functional model of metacognition, which highlighted the role of metacognitive feelings in the monitoring of cognitive activity. Since the turn of the century, studies by Metcalfe (2009), A. Schwartz (2002), Koriat (1993, 1997, 2000, 2012), and Paynter et al. (2009) have established a renewed interest in metacognitive feelings. Recent work on the topic attests to how important understanding metacognitive feelings has become for a number of research areas, including decision-making (Gambetti et al., 2020), consciousness (Norman et al., 2010), creativity (Puente-Díaz et al., 2021), education (Reber & Greifeneder, 2017), and psychopathology (Bruno et al., 2012).

To get a better picture of what metacognitive feelings are, we start by introducing two feelings that are widely regarded as metacognitive: the FoK (Koriat, 2000) and the feeling of confidence (FoC; Winman et al., 2005; Yeung & Summerfield, 2012). Table 1 offers an exhaustive list of the metacognitive feelings postulated in previous studies across psychology, sociology, education, philosophy, and neuroscience.

**Table 1.** List of Candidate Metacognitive Feelings Together With Corresponding Studies

Metacognitive feeling	References
Aesthetic experience	Dokic (2016)
Confusion	Vazard & Audrin (2022)
Déjà vu	A. S. Brown (2003)
Disorientation	Fernandez Velasco & Casati (2021a, 2021b)
Feeling of certainty	Tormala (2016)
Feeling of change in the visual field	Rensink (2004)
Feeling of coherence	Topolinski & Strack (2009a)
Feeling of competence	Bjork & Bjork (1992)
Feeling of confidence	Molenberghs et al. (2016)
Feeling of difficulty	Efklides (2002)
Feeling of familiarity	Whittlesea et al. (2001a, 2001b)
Feeling of forgetting	Arango-Muñoz (2013); Halamish et al. (2011)
Feeling of knowing	Koriat (2000)
Feeling of learning ease	Koriat (1997)
Feeling of pastness	Perrin et al. (2020)
Feeling of rationality	James (1879)
Feeling of reorientation	Charalambous et al. (2021)
Feeling of rightness	Stewart et al. (2023)
Feeling of satisfaction	Efklides (2002)
Aha experience	Bowden et al. (2005); Kounios & Beeman (2014)
Intuition	Loev (2022a)
Surprise	Reisenzein (2000)
Tip-of-the-tongue experience	S. R. Brown (2000)

Note: This list is not exhaustive, and some of the listed subjective states are more paradigmatic metacognitive feelings (e.g., feeling of knowing) than others (e.g., feeling of reorientation). There are two criteria for inclusion. First, the authors in the cited sources have referred to the phenomenon as a “metacognitive feeling” (or using an analogous term such as “noetic feeling” or “epistemic emotion”). Second, the phenomenon accords to the key aspects of metacognitive feelings: They are conscious, they have phenomenal valence, and they concern the subject’s cognitive capacities and processes. Future research might use the current account to discern which of the above candidates are bona fide metacognitive feelings. It is also possible that there is some overlap between some of the metacognitive feelings listed in this table. For instance, the feeling of reorientation after one has lost their way might not be sui generis but rather a type of insight, or aha experience, applied to the navigational domain.

For the FoK, consider the British TV program *University Challenge*, in which teams from two different universities or colleges compete to respond to a series of trivia questions. The challenge is twofold: to answer correctly and to do so before the other team. If participants feel they know the answer, they have to push the buzzer faster than the other team, with participants often pushing the buzzer before the question itself is finished. With questions as intricate as “In family relationships, what four-word term is used to describe a child of the child of a first cousin of either of one’s parents in relation to oneself?” participants often must rely on their “gut feelings” to decide whether to push the buzzer or not because if they wait until they are sure that they know the answer, the other team will push the buzzer before them (and in case you are wondering, the answer is “second cousin once removed”). The gut feeling in question is the FoK. On hearing the question, a feeling of knowing the answer emerges often before the answer itself, prompting the participant to push the buzzer.

As for the FoC, this is the kind of feeling that guides people taking a multiple-choice test in which wrong answers are penalized with negative points. The examinee might think that answer “a” is more likely than the other three possibilities, but whether they choose to select it or to leave the question blank will be influenced by how sure they are of the answer. If they feel confident that “a” is the right answer, then they will be more likely to select it instead of leaving the question blank. The feeling guiding their behavior here is the FoC.

### Key aspects of metacognitive feelings

In what follows, we outline some important characteristics of metacognitive feelings. We defend an understanding of metacognitive feelings as affective experiences (Loev, 2022b). To say that metacognitive feelings are affective is to say that their phenomenology comprises a positive or negative affect or valence (Carruthers, 2017; Proust, 2015; Russell, 1980, 2003; Topolinski & Strack, 2009c); that is, they feel positive (e.g., FoK) or negative (e.g., ToT). This component can be seen in part as an evaluation of the quality of the mental capacity or process with which the cognitive feeling in question is concerned. It is worth emphasizing that we consider valence here a phenomenal quality (i.e., to *felt* or *experienced* valence). This quality often, but not always, correlates with closely associated but ultimately nonphenomenal properties such as *object* valence (Charland, 2005).

When we say that metacognitive feelings are affective experiences, we also mean that they are conscious experiences (Koriat, 2000; Koriat & Levy-Sadot, 1999). Here, it is useful to distinguish between phenomenal and access consciousness (Block, 1995). Phenomenal consciousness is subjective experience, the “what it is like” to be in a given state. Access consciousness, in contrast, is more restrictive and refers to those aspects of conscious experience that we can consciously report to ourselves and to others (for a review, see Overgaard, 2018; for recent empirical evidence that process and access consciousness are not only conceptually distinct but can also be teased apart empirically, see Amir et al., 2023). With this distinction in mind, we can say that metacognitive feelings can at times be unconscious in the sense that they are not directly accessible, and yet, we take them to be phenomenally conscious in that their emergence makes a phenomenal difference and shapes conscious experience. This is also in line with previous work arguing that metacognitive feelings often reside on the fringe of consciousness (James, 1890; Mangan, 1993, 2000, 2001; Norman et al., 2010; Reber et al., 2002) and may sometimes be considered “background feelings” (Colombetti, 2011, 2014).

The evidence that metacognitive feelings are valenced affective experiences comes primarily from findings that they covary with bodily and behavioral markers of valence. Topolinski and Strack (2009a), for instance, presented subjects with word triads that were either coherent (e.g., “salt,” “deep,” and “foam,” implying “sea”) or incoherent (e.g., “dream,” “ball,” and “book”). Coherent triads corresponded to increase activation of the smiling muscle, zygomaticus major, and increased inhibition of the frowning muscle, corrugator supercilia, patterns generally regarded as symptomatic of positive affect (Larsen et al., 2003). Metacognitive feelings have also been shown to lead to increased liking, a behavioral measure of positive valence (Forster et al., 2016; Topolinski & Strack, 2009a, 2009b; Trippas et al., 2016; Winkielman et al., 2003). Another source of evidence comes from misattribution studies. First, subjects misattribute nonaffective epistemic properties (e.g., familiarity, coherence, grammaticality) based on affective manipulations (Baudouin et al., 2000; Duke et al., 2014; Garcia-Marques et al., 2004; Lander & Metcalfe, 2007; Monin, 2003; Phaf & Rotteveel, 2005; Topolinski & Strack, 2009c). Second, in other studies, informative affective reactions are discounted by being misattributed to an irrelevant source (Topolinski & Strack, 2009a, 2009b). Together, these findings demonstrate that the valenced experience of metacognitive feelings serves to evaluate cognitive processes.

The above are all characteristics that metacognitive feelings share with all affective states. All affective states are experiences that involve valence. As for the particular function of metacognitive feelings, Arango-Muñoz and Michaelian (2014) made the following suggestion: A subject lacks direct access to and thus cannot know for certain their own mental capacities and processes. The role of metacognitive feelings might be to enable the subject to deal with this uncertainty of the mind (Arango-Muñoz & Michaelian, 2014). Metacognitive feelings concern the subject’s own mental or cognitive capacities and processes. This is essentially why they are called metacognitive feelings. For instance, the content of the FoK could be redescribed as “I know this” (i.e., as informing the subject about their ability to undertake a given mental task such as memory retrieval; Dokic, 2012). A further important characteristic of metacognitive feelings is that they motivate and influence the subject’s bodily and, importantly, mental behavior and actions in characteristic ways (Norman et al., 2010). The FoK might prompt a given participant of *University Challenge* to press the buzzer, and the *feeling of forgetting* might induce a subject to try to remember whatever it is they might be forgetting by, for example, mentally going through all of the objects they need to pack before leaving the house (Arango-Muñoz, 2013; Halamish et al., 2011). Koriat (2006) put the link between metacognitive feelings and action in causal terms: Current research in metacognition operates under the assumption that metacognitive feelings play a causal role in influencing behavior.

These considerations concerning the involvement of metacognitive feelings in the management of cognitive uncertainty and (mental) behavior reflect well a common theme within the literature: Metacognitive feelings are considered part of metacognitive monitoring and control (i.e., they stem from mechanisms that monitor and control cognition; for a review, see Proust, 2014). Intuitively, this fits well with the previously outlined affective nature of metacognitive feelings, which is characterized by valence. Valence can be understood as a monitoring-based evaluation of the current activity of the system (Proust, 2015). This evaluation results in the allocation of relevant resources to said activity. These events characteristically modify the system’s physical and mental processes (i.e., they exert control).

### Current views of metacognitive feelings

Most of the existing models in the psychological literature either encompass an entire aspect of metacognition (e.g., decision-making; Fleming & Daw, 2017) or focus on a single metacognitive feeling, such as the feeling of familiarity (Whittlesea, & Williams, 2001) or the feeling of certainty (Navajas et al., 2016). However, we can consider how a given framework of metacognition applies to metacognitive feelings, or how a particular model of, for instance, the FoC, extrapolates to other metacognitive feelings.

The dominant view in early approaches to the study of metacognition was the direct-access model (R. Brown & McNeill, 1966; Hart, 1965). According to direct-access models, there is a subpersonal monitoring mechanism that evaluates cognitive activity and induces metacognitive feelings when a particular mental state or process is detected. For example, if the monitoring mechanism detects an error in a cognitive process, this would cause a feeling of error to emerge. In the 1990s, direct-access models were heavily criticized and largely abandoned (Koriat, 1993, 1997; Reder, 1987; Reder & Ritter, 1992). They were particularly hard-pressed to account for studies that showed the confabulatory nature of many self-reported metacognitive judgments (for a review, see Carruthers, 2009).

Direct-access models gave way to heuristic-based views, according to which a series of heuristics dictated the emergence of metacognitive feelings. For example, FoKs are partly determined by familiarity with question terms (Reder & Ritter, 1992) or the accessibility of partial information (Koriat & Levy-Sadot, 1999). The feeling of familiarity depends on the perceptual fluency in the processing of a stimulus, which serves as a cue for previous encounters with it (Oppenheimer, 2008; Whittlesea & Williams, 1998, 2001a, 2001b). The emergence of the feeling of familiarity can also be affected by the manipulation of the properties of a stimulus’s size and clarity (Kelley & Rhodes, 2002; Rhodes & Castel, 2008). Similar heuristics also apply to other metacognitive feelings (e.g., the ToT experience; A. Schwartz, 2002; B. L. Schwartz & Metcalfe, 2011).

Recent developments concern the role that embodiment plays in the heuristics underlying metacognitive feelings. Goldinger and Hansen (2005) tasked participants with a recognition test, and in half the trials, a subliminal vibration underneath their seat coincided with the stimulus onset. The vibration increased the likelihood of participants responding “old” both correctly and incorrectly. This line of findings also extends to interoception: More recent work has shown that cardiovascular feedback influences recognition judgments (Fiacconi et al., 2016), and unexpected arousal induced by unseen disgust cues modified participant’s sense of confidence in a motion-discrimination task (Allen et al., 2016). The evidence outlined above suggests that our understanding of metacognitive feelings should be grounded in a somatic understanding of emotions more generally (Dokic, 2012). Affective states recruit bodily signals to model the current state of affairs (Critchley & Garfinkel, 2017; Prinz, 2004). In the case at hand, somatic cues serve as heuristics for the state of the subject’s cognitive processes.

Different computational models have emerged to account for different aspects of metacognition that are relevant to the study of metacognitive feelings. Regarding confidence in decision-making (or perception), many models rely on the feed-forward monitoring of decision (or sensory) evidence. Ballistic accumulation models postulate a confidence threshold based on the speed of evidence accumulation (Kiani et al., 2014; Kiani & Shadlen, 2009), and signal-detection models postulate a confidence threshold based on the intensity of stimuli (Galvin et al., 2003; Maniscalco & Lau, 2012). An issue for feed-forward monitoring models is that some empirical manipulations influence confidence judgments without altering choice accuracy (Bang et al., 2019; Boldt et al., 2017; Fleming et al., 2015; Wokke et al., 2017; Zylberberg et al., 2012). Hierarchical accounts, according to which metacognitive processes are second-order monitors of the evidence emerging from a first-order decision system, are better poised to account for the divergence between confidence and choice accuracy (Bang et al., 2019; Fleming & Daw, 2017; Pasquali et al., 2010).

At the heart of the rivalry between hierarchical and feed-forward models of metacognition lies the tension between parsimony and explanatory power. An advantage of feed-forward models is that performance monitoring and decision-making arise from the same computational process: evidence accumulation. An advantage of the hierarchical models is that they are better at explaining how choice accuracy and confidence judgments can come apart (for a recent discussion, see Desender et al., 2021). In the current contribution, we use the PP framework to postulate a single mechanism underlying first-order cognitive processes and metacognitive feelings, and we show how this mechanism can account for the existing empirical evidence.

## Predictive Processing

A relatively recent development in the cognitive sciences is the emergence of PP, a theoretical framework that conceives the brain as a dynamic and hierarchical prediction engine. PP has its origins in the predictive coding of computer-science models as an efficient technique for compressing data (Atal, 2006; Elias, 1955). Within the realm of neuroscience and psychology, PP offers an account of brain function that is metabolically efficient (Friston, 2010; Rao & Ballard, 1999; Sterling & Laughlin, 2015) and neurobiologically plausible (Bastos et al., 2012). The central tenet of PP is that, using previous information about the world, the brain makes predictions about it. These predictions go from the top down (i.e., from abstract levels down to sensory organs and effectors) and sideways. In turn, the errors arising from these predictions (i.e., prediction errors, or PEs) go sideways and from the bottom up and are used to update the system’s predictions in a continuous feedback loop. Over time, the overarching goal of the system is to minimize PE.

One of the chief attractive features of the framework is its unificatory potential (Clark, 2013). The ambition of many PP proponents is that a diversity of aspects of cognition can all be accounted for as part of a unified process of prediction optimization. This unificatory potential is also a key reason for using PP in the current account. PP offers a way of accounting for feelings and for mental action using a single theoretical framework. Within the theoretical toolbox of PP, we find the tools to construct an understanding of how metacognitive feelings emerge and guide action in the face of cognitive uncertainty.

In PP, perception then becomes not a bottom-up but a top-down process of continuously explaining away PE to successfully infer the hidden causes of changes in sensory input. Each layer in the hierarchy tries to predict the activity of the layer below using models that develop to capture regularities in the variation of sensory signals. Sensory sheets capture the mismatch between this cascade of predictions and incoming sensory input (i.e., PE) and the PE that cannot be explained away solely by lower layers adapting their predictions travels upward in the hierarchy. As a result, each level of the hierarchy tracks regularities at larger temporal and spatial scales than the level below it. There is widespread evidence of predictive sensory processing across most domains, from visual (Alink et al., 2010; den Ouden et al., 2010; Egner et al., 2010; Kok et al., 2012) to auditory (Blank & Davis, 2016; Wacongne et al., 2012) to somatosensory (Shipp et al., 2013; Yu et al., 2019).

PE is reduced over time both by making more accurate predictions about the world and by acting on the world to fulfill predictions. Action occurs to fulfill emergent predictions. For example, for a subject to reach for a glass of water, the system predicts (at a high level) the required movement to reach for the glass, and a cascade of descending proprioceptive predictions ensue (e.g., predictions about the movement of the body to reach the table, then of the arm, then the hand). If the subject failed to make the required movement, there would be a substantial increase in PE. The movement, then, occurs to reduce the PE of the predictions corresponding to the reaching of the glass of water.

As we have seen, both action and perception are part of the same process of reducing PE over time. In the version of PP that we follow in this article, we conceive of this process in terms of active inference. One of the upshots of the theory of active inference is that, by considering that actions fulfill predictions, it separates the problems of optimizing action and perception (Friston et al., 2016). An important element lurking in the background here is the free-energy principle. Under a series of assumptions (namely, ergodicity, plus a Markov blanket that separates internal and external states), free energy—an information theoretical measure that decomposes into complexity and accuracy—places an upper bound on the entropy of sensory states. Minimizing free energy therefore provides a tractable way for a system to approximate Bayesian inference. The free-energy principle states that organisms minimize the free energy of their internal states, which ensures that they resist the natural tendency toward disorder. Most of our discussion is phrased in terms of predictions and PE minimization (i.e., not in terms of free energy), but it is important to note the elements of information theory underlying the version of PP used here.

### Precision weighting and mental action

**Fig. 1.**
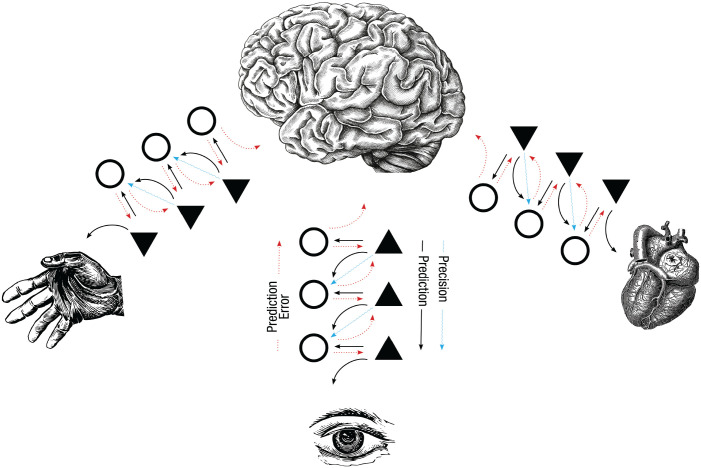
Schematic representation of the hierarchical model of brain function advanced by the predictive-processing framework. Predictions (priors) are represented as black lines cascading down the hierarchical levels from prediction units, which are shown as triangles. Prediction errors (PEs) are represented as red dotted lines climbing up the levels of the hierarchy from PE units, which are shown as circles. The straight arrows signal local processing within a level involving both PEs and predictions, which occur at every level of the hierarchy. Expected precision, which serves as a mechanism weighting PEs versus the priors, goes down the hierarchy and is represented as a blue wavy arrow. The three icons at the bottom of the hierarchy represent proprioception (left), exteroception (center), and interoception (right). Percepts and actions occur when PEs are minimized at all levels within the hierarchy.

According to PP, PE serves to constantly update predictions, but not all PEs are equally reliable. For example, the PE coming from stimuli with a low level of noise (e.g., a clear road during the day) is more reliable than the PE coming from stimuli with a high level of noise (e.g., a foggy road at night) because the PE of the former is relatively less likely to be due to noise than to the inaccuracy of the current prediction. Consequently, not all PEs have the same weight when it comes to updating hypotheses about the world. The errors coming from sources that are expected to have a low variance are assigned a larger weight compared with the errors coming from sources that are expected to have a high variance. This weighting process biases the competition between information coming from different modalities (e.g., lower gain for visual signals from a foggy road at night) as well as the competition between incoming sensory inference and top-down predictions (e.g., higher gain is assigned to the remembered outline of the road’s curvature when driving on a foggy night than when driving on a clear day). The inverse of variance is called precision, and the brain, in addition to first-order predictions, is constantly trying to estimate the precision of forthcoming PEs (see Fig. 1).

Neurobiologically, current models theorize that expected precision is instantiated through synaptic gain-control mechanisms that use neuromodulators such as dopamine (Fiorillo et al., 2008; Galea et al., 2012; Iglesias et al., 2021). Recent research has related dysfunctions in precision weighing to a host of psychological disorders, such as maladaptive stress (Krupnik, 2020; Linson et al., 2020), depression (Kube et al., 2020), psychotic hallucinations (Corlett et al., 2019; Sterzer et al., 2018), and posttraumatic stress disorder (Wilkinson et al., 2017).

Derivatively, increasing precision over time is conducive to reducing PE over time. Thus, when the system is choosing among a variety of hypotheses, it is not only weighting how much PE different hypotheses are expected to generate but also the expected precision that each hypothesis is expected to generate. Accordingly, the brain is always trying to optimize precision over time, mainly by sampling the stimuli that are predicted to have high precision, which is conducive to PE minimization over time. Modulating precision weightings is also a way for the system to adapt to context in a flexible way. In the PP literature, this process of precision optimization is what defines attention (Feldman & Friston, 2010; Hohwy, 2012). Importantly, for the purpose of the current article, flexible precision weighting is also relevant for understanding action within the PP framework: The selection of particular action policies involves assigning low precision to sensory information about the current status of the body, which allows the proprioceptive predictions consistent with the desired bodily trajectory to prevail (Clark, 2020; Pezzulo, 2012).

An important subset of actions are epistemic actions (i.e., as opposed to instrumental actions), which serve to acquire better information for making future predictions (Friston et al., 2015). The paradigmatic example of an epistemic action is foraging, when a subject explores a novel setting in the hope of building more accurate generative models. Epistemic actions can take form both externally (e.g., by exploring an environment) and internally (e.g., by simulating the outcome of possible actions; Pezzulo, 2017). This is key to understanding mental action, which in PP is conceived of as a type of epistemic action. The idea is that mental action is aimed at increasing epistemic value, which is the expected information gain according to predicted outcomes (Friston et al., 2015). In PP, mental action then becomes “the predictive control of effective connectivity aimed at optimizing the epistemic value of attentional and cognitive states” (Metzinger, 2017, p. 17).

The idea is that mental action predicts a certain epistemic gain (e.g., finding out what the square root of 36 is), and, through changes in precision weighting, a novel pattern of connectivity across different brain regions emerges to fulfill the prediction of epistemic gain. This is directly analogous to how nonmental action consists of proprioceptive predictions that, through precision weighting, result in a series of bodily movements. In other words, mental action is a form of policy selection over higher level cognitive states (Sandved-Smith et al., 2021). Achieving epistemic goal states leads to the reduction of uncertainty and to PE minimization. This achievement requires cognitive control over one’s cognitive processes, which involves monitoring and precision-modulation mechanisms that are analogous to those involved in overt action control (Pezzulo, 2017).

### Affective experience

Because the central topic of this article concerns metacognitive feelings, let us now turn to theories of affective experience within the PP framework. There are two families of theories of emotion within PP that are compatible but focus on different levels of analysis. One family of theories focuses on interoception and (in its most recent rendering) posits that emotion emerges out of the regulation and control of interoceptive variables (Barrett & Simmons, 2015; Gu et al., 2013; Seth, 2013; Seth & Friston, 2016; Seth & Tsakiris, 2018; for a precursor, see Damasio, 1994). Another family of theories focuses on PE dynamics and posits that affective dimensions correspond to informational aspects of PE reduction (Fernandez Velasco & Loev, 2021; Hesp et al., 2021; Joffily & Coricelli, 2013; Van de Cruys, 2017; Van de Cruys & Wagemans, 2011). Although their focus is different, the two positions are not only compatible but also complementary. The former family of theories provides a detailed mechanism of interoceptive inference, and the latter provides a computational equivalent of valence.

Interoception refers to the perception and control of the visceral cycles (Barrett & Simmons, 2015). Interoception ranges from sensory information about heartbeat, breathing, stomach activity, and so on, to visceromotor signals and allostatic reflex arcs. Interoceptive inference refers to the regulation of interoception through the use of a deep generative model (Seth & Friston, 2016). The aim of interoceptive inference is to attain not only homeostasis (maintaining a metabolically steady state) but also allostasis, which is the anticipatory control of homeostatic needs (Sennesh et al., 2022). Maintaining homeostasis involves only low-level adjustments, but allostasis involves longer timescales and thus deeper generative models. Interoceptive inference operates within a larger dynamic system involving other aspects of cognition, as supported by a fast-growing body of evidence showing the impact of interoceptive processes on perception and metacognition (Allen et al., 2016; Garfinkel et al., 2014; Hauser et al., 2017; Salomon et al., 2016). It is this longer timescale, higher scale process of interoceptive inference, that gives rise to affective experience (Seth & Friston, 2016; Seth & Tsakiris, 2018). In terms of neuroanatomy, interoceptive inference is mapped across a network involving the amygdala, the anterior cingulate, and the anterior and posterior insula. The amygdala receives exteroceptive and interoceptive information and interacts directly with the anterior insula and with the posterior insula through neuromodulation. The anterior cingulate monitors and controls the precision of ascending visceral information via neuromodulation. Finally, the anterior insula is hypothesized to play a computational role in autonomic policy selection (for a more detailed picture, see Allen et al., 2022).

As for PE dynamics, the most recent computational account within PP characterizes valence as resulting from fluctuations in the estimated confidence an agent has in their generative model of the world (Hesp et al., 2021). Crucially, different valence values result in different patterns of modulation of action selection. Hesp and colleagues cast their model in terms that are germane to the free-energy principle, so that the agent infers subjective fitness by estimating the precision of the relevant action policies. Now, the rate at which PE is minimized is partially determined by the precision ascribed to the corresponding prediction, so that if an organism holds expectations about precision, it implicitly holds expectations about the rate of PE reduction by extension (Perrykkad & Hohwy, 2020). Therefore, in terms of PE, we can conceive positive phenomenal valence as equivalent to a positive expected PE reduction rate (ExPERR). Likewise, we can conceive negative phenomenal valence as equivalent to a negative ExPERR. Note also that the ExPERR is a better candidate for phenomenal valence than simply the PE rate because, paradigmatically, predictions but not PEs form the content of conscious experience in the PP framework (Chanes & Barrett, 2016; Hohwy, 2012). Earlier contributions (e.g., Joffily & Coricelli, 2013; Van de Cruys, 2017) followed an understanding of valence as the PE rate. This is a problem if we want to account for phenomenal as opposed to unconscious valence (Fernandez Velasco & Loev, 2021). The more recent accounts are better equipped to explain phenomenal valence, particularly if we want to do justice to the idea that metacognitive feelings are conscious experiences.

The integrative view of interoceptive and computational accounts depicts a system of interoceptive inference in which expectations about PE dynamics play a key role in the monitoring and control of allostasis. Different types of emotions correspond to different affective models, or categorizations, of a situation, and they serve to maintain homeostasis, guide action, and mold perception (Barrett, 2017). In the PP view, feelings are holistic models that are both descriptive (i.e., evaluative) and directive (i.e., action policies). PP theories of emotion strengthen the connection between valence and action. In a similar vein, Kiverstein et al. (2019) argued that in PP valence is inherently action-oriented so that “at the same time as emotional experiences feel good or bad, they also prepare or make us ready to act on relevant affordances” (p. 2858). Because the ExPERR (as opposed to simply the PE rate) depends on deep temporal models, valence can influence action so as to guide the organism to an overall reduction of uncertainty over time (Hesp et al., 2021).

To unpack the PP perspective on affective experience, let us consider the classic bear-in-the-forest scenario introduced by William James (1884). If a bear appears, a prediction of increasing PE (i.e., negative ExPERR) results in negative felt valence. The bear is inferred to be the cause of the predicted PE dynamics. An affective model of the situation emerges, both evaluating it (e.g., there is a high likelihood of damage) and regulating it (e.g., prompting the subject to run away). In the resulting picture, affective experiences transform the subject’s action policies in favor of certain behaviors, and the function of the feeling is to model the situation and its link to error dynamics so that action can emerge in a regulative fashion.

## A PP Perspective of Metacognitive Feelings

The current work aims to explain how metacognitive feelings arise and how they affect behavior—mental behavior in particular. Let us first address some potential sources of confusion. According to the PP framework, prediction is the basic form of cognition, or cognitive processing. However, all feelings are conscious, and being conscious entails in PP a prediction of some sort. In this sense not only perception, action, attention, and so on but also *all* feelings can be considered cognition. This is similar to the inclusive notion of “cognition” as information processing in cognitive science (Neisser, 1967). In addition, all affective feelings, having a valence understood in the way outlined above, are about predictions (i.e., cognition) or, more precisely, prediction dynamics. In this sense, and similar to attention, they can all be considered a form of metacognition (Van de Cruys, 2017, p. 10; see also Carruthers, 2017).

What distinguishes metacognitive feelings from other affective feelings is that they are cognitive (or metacognitive) in a more restrictive sense. They are cognitive by being specifically about the cognitive domain, which is traditionally characterized as comprising (with a philosophical flavor) thought, judgment, beliefs, concept use and (with a psychological flavor) memory, planning, decision-making, cognitive control, and the subpersonal cognitive processes implementing these kinds of cognition. This traditional notion of “cognitive” also tries to contrast with the terms sensory, motoric, bodily, or affective. We could now leave it at that and let the “cognitive” in metacognitive feelings be just that: being about the cognitive domain classically conceived while leaving it to readers to figure out what exactly they consider cognitive in the classic sense. We want, however, to provide a speculative extension. The PP framework provides a more rigorous way to frame the idea of the cognitive domain traditionally conceived. In PP, a system operates in various predictive modalities (i.e., there are various dynamic areas in need of modeling and prediction, of which the world and the body are prominent ones. Each area will have their own deep hierarchical generative models tailored to the specifics and regularities of the domain. For instance, exteroception and interoception with their differentiated modalities grossly comprise the sensory domain that is concerned with modeling and predicting the external world and the body, respectively. Our suggestion is that we can understand the cognitive domain in a similar way. But what is it that is modeled by classical cognition? In Metzinger’s words: “What parts of the world can be accessed by *neither* exteroceptive *nor* interoceptive predictive processing? . . . One general answer is: the brain itself; the neural body” (Metzinger, 2017, p. 16). Following Metzinger’s line of thought, we consider that the cognitive domain is best understood as the effort of the cognitive system to predict itself. What belongs to the cognitive domain are those kinds of cognition that happen when the cognitive system predicts itself.

An important insight that connects PP with empirical research on metacognition concerns the role and nature of processing fluency. The subpersonal property of processing fluency (or disfluency) is often considered to be the main proximal cause of positively (or negatively) valenced metacognitive feelings (e.g., Winkielman et al., 2003). In PP terms, processing fluency can be reconsidered in terms of the ExPERR associated with a cognitive process because if (in line with fluency accounts of valence) the organism monitors the fluency of the information processing of a given cognitive activity, changes in said fluency will result in changes in prediction success, so that if there is an increase in fluency the organism can expect, all else being equal, a proportional increase in the ExPERR.

For a demonstration, please read the following sentence: “The haystack was important because the cloth ripped.” You will probably feel confused, unable to understand (Auble et al., 1979). This is (hopefully) in stark contrast to how you felt about the previous paragraphs, which you mostly understood. The processing responsible for parsing the text unexpectedly turns disfluent. In other words, the parsing process suddenly becomes a potent source of PE, making the processing-specific ExPERR drop. Relative to the parsing process this leads to the prediction of a highly negative ExPERR (negative valence), which is to say it leads to a negative cognitive feeling: a feeling of confusion, incomprehension, or not understanding. Now try to attend to what happens when we give you the following hint: parachute.

You likely feel much better now than a few seconds ago. This makes sense: The processing responsible for parsing the text, previously highly disfluent, unexpectedly turned fluent. In other words, the parsing process, previously a potent source of PE, eliminated a big chunk of PE in one quick sweep, making the actual processing-specific ExPERR spike. Relative to the parsing process this leads to the prediction of a highly positive ExPERR (positive valence), leading to a positive cognitive feeling: the feeling of understanding, sometimes also called the aha or eureka experience (Bowden et al., 2005; Gopnik, 1998; Trout, 2002). Such an analysis of proximal causes in terms of changes in processing fluency or the processing-specific ExPERR can be generalized to all affective feelings. By conceiving of *phenomenal* valence as the ExPERR, we can see both how metacognitive feelings are conscious—because valence, as a prediction, becomes part of conscious experience—and how experienced valence is part of an evaluative process—because valence emerges from evaluating the expected increase in PE of a given cognitive process. This is in line with an understanding of experienced fluency not in absolute terms but in relative terms of changes in fluency, with expectations playing an important role (Wänke & Hansen, 2015). It is also in line with the “feelings-as-information” theory, which proposes that people attend to their feelings as a source of information in a flexible and context-sensitive way (Schwarz, 1990, 2012) and that has recently been applied to metacognitive feelings (Schwarz et al., 2021). What PP does is to reconceive both perceptual (Reber & Schwarz, 2001) and conceptual (Zhang & Schwarz, 2020) fluency in terms of predictive dynamics (Brouillet & Friston, 2023).

### Metacognitive feelings and mental action

We have seen in the previous section that valence will result in affective generative models. In the case of the FoK, expected success in retrieving a certain information (e.g., the answer to the question “What is the capital of Nepal?”) will lead to an increase in the ExPERR and to the experience of positive valence directed at the process of retrieval (i.e., the process required to answer the question) in the form of a model that predicts imminent information retrieval, corresponding to the experienced sense of knowing the answer to the question. The subject would thus take their FoK not only to be about recalling the capital of Nepal but also about their knowledge of it or their ability to recall it. Note that in the subject’s phenomenal experience, the feeling might be about a process (answering the question) or about the content at which that process is directed (the capital of Nepal, which appears as about to be revealed). Interestingly, it is theoretically possible that the ExPERR differs depending on the source, leading to mixed, or unstable, feelings.

In most cases, the metacognitive feeling will seem to the subject to be directed to the content rather than to the process itself (Rosenthal, 2000). This is because the content is usually consciously accessible and the underlying process is not (Metzinger, 2003). Sometimes the subject might not be (immediately) aware of what their feeling is directed at because the processes and properties that give rise to it are not consciously available or because the system fails in automatically identifying and binding the affective components appropriately (e.g., to model the causes of expected changes in PE dynamics). In some cases, this will result in reiterations of the identification stage, possibly on a high level. In other words, sometimes the subject will have to resort to conscious interpretation to specify their metacognitive feelings.

Concerning this issue, there is substantial empirical evidence that points to the importance of the perceived context in determining the nature of metacognitive feelings (Koriat et al., 2004). For example, in a learning experiment, framing questions in terms of forgetting (in contrast to remembering) reduced the subjects’ confidence (Finn, 2010). Interpreting these results from the PP viewpoint; the way the subject frames the situation changes the resulting metacognitive feeling. If the subject directs their attention to forgetting, all of the elements that might support the hypothesis that the subject is forgetting something will have a higher weight when the system generates a model of PE dynamics, making the emergence of a feeling of forgetting more likely (resulting in an expected increase in PE and thus negative valence). In contrast, if the subject directs their attention to remembering, the elements that might support the hypothesis that the subject will remember will have a higher weight when the system generates a model of PE dynamics, making the emergence of an FoK more likely. As with all affective experiences, valence will constitute the core of the feeling, which will serve to frame (i.e., model) the situation in a dynamic, affective fashion.

**Fig. 2.**
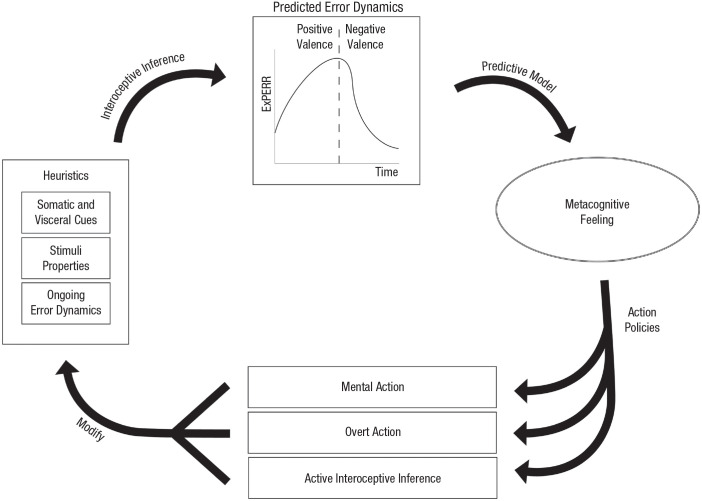
Schematic representation of the proposed predictive processing account of metacognitive feelings. Interoceptive changes, properties of the stimulus, and current prediction-error (PE) dynamics serve as heuristics for predicting PE dynamics associated with a target cognitive process. If the expected rate of PE reduction is positive, then positive valence ensues. If the expected rate of PE reduction is negative, then negative valence ensues. Metacognitive feelings arise on the basis of the predicted dynamics of PE in the form of a holistic, dynamic model. The model is both descriptive and directive. It corresponds not only to an appraisal of the situation but also to particular action policies. These action policies cascade into mental action (e.g., continuing or stopping the target cognitive process), overt action (e.g., use of a cognitive artifact), and interoceptive changes (e.g., increased heartbeat). The action policies result in a transformation of the overall situation, including both somatic states and incoming PE. The result is an iterative complex of activity in which metacognitive feelings evaluate and regulate cognitive processes in a dynamic fashion.

As we saw in the previous section, we should understand feelings not only as descriptive models of PE dynamics but also as action policies. The result is that when feelings emerge, they transform the subject’s action policies and influence the subject’s navigation through them. As for metacognitive feelings, we know that they influence not only physical but also mental action. According to our account, the process of precision weighting involved in a cognitive process implicitly involves the generation of the ExPERR, which corresponds to phenomenal valence directed at that process. A metacognitive feeling (with valence as one of its core dimensions) then emerges to model the cognitive process in question. Crucially, the tight link between valence and action (explored in the previous section) means that a metacognitive feeling also alters the weighting of potential mental-action policies. It models the cognitive domain in both an evaluative and *regulative* fashion so that it biases the competition between different cognitive affordances. In other words, a cognitive feeling makes certain mental actions (e.g., instances of remembering, calculating, reasoning, or imagining) more probable than others. The idea that metacognitive feelings express mental or cognitive affordances in a nonconceptual way has been defended (Proust, 2009b), and PP gives us a framework to clarify how this works: Metacognitive feelings bias the competition between different cognitive action policies, which in turn influences the probability of occurrence of different mental actions (see Fig. 2).

To illustrate the proposed account, picture a situation in which a subject is asked what the capital of Nepal is. The subject does not immediately come up with an answer, but an FoK emerges. This FoK modifies the subject’s cognitive affordances, making it more likely for the subject to undertake mental actions related to retrieving the name of the capital of Nepal (see also Arango-Muñoz, 2013; Michaelian, 2012). A lot of different actions would be positively biased in this way, such as imagining where Nepal is on the map or remembering the capitals of different countries in the region, or, more simply, maintaining the cognitive process of information retrieval. When it comes to the system making predictions to mentally act, these possible actions will have become more likely to be selected and to be allocated the cognitive resources necessary to perform the mental actions. This biasing of cognitive affordances happens at different levels and timescales (Jorba, 2020; McClelland, 2020). It is likely that not only immediate mental actions but also distal predictions such as goals will become more probable because of metacognitive feelings (see Pezzulo & Cisek, 2016). Back to our example, the FoK will make the hypothesis that the subject knows what the capital of Nepal is more likely and the goal of retrieving the name of the capital of Nepal more likely to be pursued.

A final point worth emphasizing is that the dynamics involved here are nonlinear. Because all of this happens in a feedback loop, metacognitive feelings will modify the likelihood of mental actions, but mental actions will also modify metacognitive feelings in turn by affecting the expected PE dynamics and with them the predictions (e.g., valence) determining the metacognitive feelings. This is in line with the emerging picture of empirical studies of metacognitive feelings, in which “monitoring drives control, and feedback from control operations then produces monitoring output, which in turn drives control, and so on” (Koriat, 2006, p. 98). In general, engaging in (mental) actions spans predictive trajectories in which course feelings can arise in a regulative fashion (Proust, 2013). What this means is that in performing the component steps that implement a mental action, PE dynamics unfold, leading to possible alterations in the expected PE, giving rise in turn to regulative metacognitive feelings in the form of predictions about PE dynamics.

To unpack this, let us go back to our subject struggling to remember the capital of Nepal. If, guided by a FoK, the subject spends a long time invested in mental activity trying to remember the capital of Nepal unsuccessfully, the hypothesis that the answer will be retrieved (and thus the goal of retrieving the answer) becomes less likely. This makes the ExPERR go down because there is mounting evidence that the subject does not actually know and that information retrieval will be unsuccessful. As a result, the positive valence wanes, eventually making the FoK dissolve and possibly transform into a ToT experience with negative valence. Conversely, if the subject remembers that the capital of Nepal is Kathmandu, the FoK will also eventually dissolve because the predicted increase in the PE reduction rate (related to the process of memory retrieval) will have taken place. The expected epistemic gain that the FoK was signaling will have already happened. The PE will have decreased on finding that the capital of Nepal is Kathmandu, thus fulfilling the goal and the corresponding prediction of the subject knowing the answer to the question. Because no further PE reduction is expected related to memory retrieval, the system will stop predicting related positive ExPERRs, and the FoK will dissipate.

### Empirical evidence and computational models

In the above, we have proposed a PP perspective of how metacognitive feelings arise and how they guide behavior. Different metacognitive feelings correspond to different models of predictive dynamics. Thanks to the current unificatory account of metacognitive feelings, specific computational models for each metacognitive feeling could then be framed within the larger PP framework. Regarding direct evidence, it is important to keep in mind that PP is still in its early stages and that the challenges to operationalize it are substantial because at the heart of the framework are hidden (i.e., unobservable) states and their dynamics that in turn depend on the learning history of each individual (Van de Cruys et al., 2022). Nevertheless, there is a growing body of evidence that aligns with the proposed PP perspective on metacognitive feelings that comes from empirical work in psychology and neuroscience, as well as some relevant computational models.

Key evidence for the proposed view comes from findings about the heuristic nature of metacognitive feelings, with the experimenters in question interpreting their findings through the PP framework. For instance, in a series of experiments on the feeling of pastness, Brouillet and colleagues (2023a, 2023b) conceptualized fluency as an estimation of the precision of predictions. This is very much in line with the proposed perspective in which estimated precision and the ExPERR go hand in hand because if an agent has expectations about precision, they implicitly have expectations about the rate of PE (Perrykkad & Hohwy, 2020). In their most recent experiment, Brouillet and colleagues (2023b) found that when there is an experienced gap between conceptual and perceptual fluency, a feeling of pastness arises. In other words, when there are conflicting sources of the ExPERR, the feeling of pastness emerges as a model of these prediction dynamics that corresponds to the inference that the stimulus in question has been encountered before. Turning to the study of surprise, Gerten and Topolinski (2019) manipulated two orthogonal factors: an event’s deviation from expectation and the event’s ease of integration with previous representations. Contrary to the previously dominant theories of surprise, they found that surprise was not well explained by these two factors. Rather, surprise was best explained as the result of the temporal interplay between perceptual input and the continuous fine-tuning of expectations, which Gerten and Topolinski interpreted in PP terms. Again, in line with the current perspective, surprise is best understood as a dynamic model of PE. Concerning the feeling of suspense, Li and colleagues (2021) designed a task based on blackjack in which a variety of suspense dynamics can be experimentally induced. The model that best explained their behavioral results was one in which suspense corresponded to the expectation that consequential information will be revealed in an upcoming moment (Ely et al., 2015). In PP terms, suspense would then be a model of upcoming prediction dynamics. In particular, it would model a sharp (and uncertain) change in the ExPERR. Finally, the heuristics model of insight from Laukkonen and colleagues is also nested in PP. In a high-powered experiment, they showed that participants rated worldview beliefs (e.g., “people’s core qualities are fixed”) as truer when they solved anagrams and experienced corresponding aha moments (Laukkonen et al., 2022). In other words, artificially induced aha experiences resulted in insight misattribution, which the authors interpreted through a PP hierarchical model: When a valuable idea is uncovered through implicit, lower level processing, it goes higher up the hierarchy, leading to sudden awareness of it. Given prior belief, the aha experience then acts as a signal of the expected epistemic gain from the idea (i.e., an ExPERR model in terms of the current account).

The misattribution of insight also touches on a theme that is key to our proposed account: mental action. We see metacognitive feelings both as descriptive and directive, which means that they transform action policies, particularly concerning mental action. In the insight misattribution, we can see that the aha experience is not epiphenomenal. Rather, it results in the action of endorsing the idea that is inferred to be at the root of the expected changes in prediction dynamics. Regarding the link between metacognitive feelings and mental action, in a study in which participants were probed with previously studied face-name pairs and subsequently provided with an opportunity to select limited pairs for restudy, there was a positive relationship between FoK ratings for unsuccessful recalls and subsequent item selection for restudy (Brooks et al., 2021). ToT experiences have likewise been linked to increased information-seeking behavior (Litman, 2005; Metcalfe et al., 2017). In contrast, items with lower judgment of learning ratings were restudied more frequently than those with higher ratings (DeCaro & Thomas, 2019). In our account, each metacognitive feeling is a different affective model, so not only will they model prediction dynamics differently, they will also each have proprietary effects on action policies.

Going back to the case of insight, it is notable that embodiment plays a key role. In a separate experiment by Laukkonen and colleagues (2021), participants indicated aha experiences through a dynamometer, which measures grip strength. The feelings strongly mapped onto the accuracy of solutions, and interestingly, participants unintentionally gripped the dynamometer more tightly during more intense insights, which further predicted the accuracy of their ideas. Interestingly, the affective component of aha experiences might also extend to perception. Sudden switches of viewpoint in bistable illusions elicit states similar to aha experiences. In a recent experiment, such switches yielded increased zygomaticus major activity, indicating increased positive affect (Lindell et al., 2022). The experimenters linked this result to a PP theory of aesthetic perception in which positive affect follows the reduction of PEs (Sarasso et al., 2020). Note that in line with more recent PP theories of affect, the effect is not explained only by error reduction but rather by epistemic gain and the corresponding decrease in expected PE. Another study of feelings about perceptual processes, this time designed to test the ideas behind interoceptive inference using disgust cues, found that unexpected arousal regulated perceptual precision, such that the experience of confidence reflected the integration of both external sensory and internal embodied states (Allen et al., 2016). In a more recent computational article, Allen and colleagues (2022) argued that their interoceptive inference model can also be extended to higher order functions that would encompass feelings about cognitive processes. An experiment by Fiacconi and colleagues (2016) points in this direction. When participants went through a recognition memory task, the experimenters found that faces presented during cardiac systole (i.e., maximal visceral feedback) were more likely to be deemed “old” than faces presented during cardiac diastole (i.e., minimum afferent feedback). This influence of afferent feedback was specific to unsuccessful recollection trials in which participants reported a feeling of familiarity. Thus, cardiovascular feedback had an effect on metacognitive feelings that the experimenters interpreted through PP interoceptive and constructivist models (Barrett & Simmons, 2015; Seth et al., 2012). These findings are in line with a broader literature suggesting a close, affect-mediated connection between feelings of familiarity and autonomic feedback (Duke et al., 2014; Harmon-Jones & Allen, 2001; Winkielman & Cacioppo, 2001). A further study showed a similar effect of visceral information for the FoK that was moderated by interoception, so that for individuals with higher interoceptive sensitivity, increases in heart rate for old items were associated with larger differences in FoK (Fiacconi et al., 2017). And Garfinkel and colleagues (2013) found that interoceptive sensitivity modulates the extent to which subjective confidence in a target-detection task predicts subsequent memory performance.

Turning to neuroscientific evidence, the PP theory of interoceptive inference hypothesizes that the insula integrates top-down predictions with ascending viscerosensory inputs to compute PEs that are then used to model affective states. Studies using innovative techniques, such as optogenetics, cellular-resolution functional imaging, and circuit tracing have given empirical support to this hypothesis (for a discussion, see Allen, 2020). Gehrlach and colleagues (2019) used optogenetics to map the neural circuits by which the posterior insular cortex integrates aversive stimuli with interoceptive and emotional states. They were able to activate or inhibit neurons in this region while rodents underwent noxious interoceptive or exteroceptive stimulation in a variety of settings. The stimulation of the posterior insular cortex resulted in both avoidant behavior and increased respiratory frequency, whereas its inhibition resulted in exploratory behavior and impaired emotional learning (i.e., erasing the association between shocks and anxious emotion behavior). The large majority of neurons in the region exhibited mixed coding of interoceptive and exteroceptive states, and a further analysis of the region’s connectivity through monosynaptic retrograde tracing suggests a hierarchical view of the insula in which multimodal sensory PEs are integrated to guide interoceptive self-inference. A different experiment by Livneh and colleagues (2020) showed that the neural populations in the insula maintain an active, anticipatory representation of visceral states, signaling not only the current state (e.g., thirst or hunger) but also future states so that they activate when presented with water or food cues in anticipation of bodily changes. Another key element in the PP mix is neuromodulation because expected precision is thought to be coded through neuromodulators such as dopamine. With regard to metacognitive feelings, dopamine-based neurotransmission is closely tied to curiosity (see Gruber & Ranganath, 2019), and a functional MRI study of the aha experience showed that it corresponds to changes in the dopaminergic midbrain (Tik et al., 2018), which the researchers linked to affective processing and to the PP work on dopamine firing as encoding expected precision (Friston et al., 2014).

In terms of computational models, there are currently PP models of specific metacognitive feelings (e.g., the aha experience), as well as PP models of aspects of cognition that are central to all metacognitive feelings, such as the links between phenomenology and mental action or the links between affective interoceptive processes and metacognitive uncertainty. In a recent article, Laukkonen and colleagues (2023) advanced a model of insight in which the aha experience operates as a heuristic that captures attention and permits fast action under uncertainty. They conceived the emergence of a new idea as restructuring via Bayesian reduction. The dopaminergic precision-weighting process then leads to the affective aspects of the phenomenology of insight, its attentional capture, and its consequences during decision-making. Of particular relevance for the current discussion is a recent agent-based computational model of subjective experience and mental action within the PP paradigm (Sandved-Smith et al., 2021). The authors simulated the regulation of mind wandering during a task that involves selective attention, showing that an agent that possesses a deep generative model exhibits the phenomenological cycles of mind wandering and focus that are associated with focused attention and mindfulness meditation practices (Lutz et al., 2008). They presented their simulation as a proof-of-concept case study that they argued could be extended to other cognitive processes and their phenomenology, such as affective experience (for computational simulations linking affective processes and action selection, see Hesp et al., 2021). Finally, another type of computational simulation that is important for the proposed account is a recent computational model of interoceptive inference in which, through simulated psychophysics, Allen et al. (2022) managed to reproduce commonly reported effects linking the cardiac cycle to affective behavior, showing as well how the attenuation of exteroceptive input by the cardiac cycle propagates to metacognitive uncertainty.

### Novelty of the proposed account

The current account is the first to apply PP to explain the mechanism underlying metacognitive feelings. We began this article by outlining three core characteristics of metacognitive feelings: Metacognitive feelings are affective experiences, the subject experiences a positive or negative valence as part of an evaluative process, and metacognitive feelings guide the subject’s behavior to deal with the uncertainty of the mind. We have seen that conceiving phenomenal valence as the ExPERR in the current account offers an explanation of the first two characteristics. In turn, the tight link of valence and action in PP explains the way that metacognitive feelings guide mental action. An affective model of the situation emerges out of the evaluation of a cognitive process and alters the weightings of the cognitive affordances that unfold before the subject. As the subject follows the chosen paths through this emerging cognitive landscape, the expected PE changes, and with it the ensuing valence, which again transforms the cognitive feeling and the overall affective hue of the larger mental process.

Early accounts of metacognitive feelings depended on direct-access views of metacognition (R. Brown & McNeill, 1966; Hart, 1965; Nelson & Narens, 1994), which could not explain the role that either somatic cues (Fiacconi et al., 2016; Goldinger & Hansen, 2005) or heuristics (Koriat & Levy-Sadot, 1999; Oppenheimer, 2008; B. L. Schwartz & Metcalfe, 2011) played in the emergence of metacognitive feelings. The current perspective shows the way in which a larger process of interoceptive inference uses somatic cues to, in the form of metacognitive feelings, model and control cognitive processes. More recent accounts of metacognition can be divided into feed-forward (Galvin et al., 2003; Kiani et al., 2014; Kiani & Shadlen, 2009) and hierarchical (Bang et al., 2019; Fleming & Daw, 2017; Maniscalco & Lau, 2012; Pasquali et al., 2010) models. The current account benefits from the advantages of each of the models. Like feed-forward models, it postulates a single computational process behind performance monitoring and decision-making (Desender et al., 2021). In our case, that process is precision weighting. Like hierarchical models, it can explain why some empirical manipulations influence confidence judgments without altering choice accuracy (Boldt et al., 2017; Fleming et al., 2015; Wokke et al., 2017; Zylberberg et al., 2012). Whereas cognitive processes and metacognitive processes both operate through precision weighting, decisions (including perceptual decisions) and confidence can be based on different sources of information. Even when they are based on the same sources of information (e.g., priors, visual stimuli, visceral changes), the precision weighting for each might differ. For example, noise in visual stimuli will affect perceptual decisions more heavily than it will affect the corresponding FoC, which depends less heavily on that source of information (Bang et al., 2019).

### Predictions

A recurrent worry regarding PP that might apply to the current account concerns its testability (Kogo & Trengove, 2015). Of course, as a theoretical framework, PP encompasses different theories, so its central aim is not to advance a series of hypotheses. However, PP can be also operationalized into a process theory (Friston et al., 2016), and specific theories developed under the PP umbrella (e.g., interoceptive inference) can advance specific hypotheses. In the case at hand, we can use our PP perspective of metacognitive feelings to generate the following predictions:

- Subliminal interoceptive changes induced through experimental manipulations will influence metacognitive feelings all across the board. More specifically, neuromodulatory gain control regulates precision globally so that unexpected changes in interoceptive states, such as heart-rate acceleration, should influence the emergence and effects of metacognitive feelings.
- The intensity and polarity of metacognitive feelings will be better predicted by gradients in performance rather than by its absolute quantities.
- The intensity of sudden metacognitive feelings (e.g., the aha experience, surprise, confusion) will correspond to the magnitude of sudden changes in expected epistemic gain.
- The unfolding of metacognitive feelings will all correlate with increased activation in a core network of interoceptive inference underlying all affective experiences. Unveiling the neural underpinnings of interoceptive inference is still in its early stages, but the current model involves regions such as the amygdala, anterior cingulate, and anterior and posterior insula (Allen et al., 2022).
- The alteration of neuromodulators pharmacologically (e.g., using haloperidol, which acts as a dopamine D2 receptor antagonist) would lead to a decoupling between metacognitive feelings (e.g., FoK), the performance of the relevant processes (e.g., time until recall), and proprietary mental actions (e.g., restudy).

### Limitations, challenges, and future directions

One of the key advantages of PP is its unifying potential, an aspect of the framework that is particularly beneficial for developing an understanding of metacognitive feelings. Nevertheless, this unifying potential of PP is also a source of criticism (Colombo & Wright, 2017; Klein, 2018). Critics argue that the brain involves a complex interplay of many processes, which refutes the need of a grand unifying hypothesis. Whether this critique is warranted is outside the scope of this article. Suffice to say that, although the critique might apply to the goal of explaining all of cognition, this article was somewhat less ambitious. Where there were separate accounts for each metacognitive feeling, our aim here was to provide a unified account for all metacognitive feelings. Within this context, the unificatory potential of PP proved itself to be particularly valuable.

It is also important to consider challenges to the current account. Most notably, there are findings that fluency effects are strongest when they are unexpected (Wänke & Hansen, 2015). In one study, participants had to judge the truth of a series of statements (Hansen et al., 2008). The perceptual fluency of the statements was manipulated through color contrast. High-fluency statements were judged as more likely to be true, but only when the high fluency corresponded to a change from previous fluency. This finding could pose a challenge for a PP account of metacognitive feelings. A series of low-fluency statements should generate the expectation of more low-fluency statements, so a sudden high-fluency statement should result in an increase in the PE, corresponding to a negative feeling, and, accordingly, to a judgment that the statement is false (or so the worry about PP goes). However, note that the authors themselves interpreted their findings as evidence that people generally monitor changes rather than absolute values because the former are more informative. This is exactly in line with the current account. An increase in fluency results in the expectation of predictive success (i.e., an increase in the ExPERR).

A similar phenomenon to unexpected changes in fluency can also be seen in aha experiences, in which a sudden change from ignorance to understanding leads to positive affect (see Skaar & Reber, 2020). In fact, the more unexpected the solution is, the stronger the aha experience (Savinova & Korovkin, 2022). In this context, remember that in the PP model of insight advanced by Laukkonen and colleagues (2023), the emergence of a new idea corresponds to a restructuring via Bayesian reduction that results in changes in the dopaminergic precision-weighting process. The Bayesian reduction corresponds to an expectation of epistemic gain (i.e., an increase in the ExPERR) that is modeled by the metacognitive feeling of insight. In these terms, the unexpectedness of the idea corresponds to the magnitude of Bayesian reduction and, by extension, of the expected epistemic gain. The key is that, in our account, feelings do not result directly from PE but from expectations of PE rates. Sudden changes in PE thus can lead to positive feelings if they result in an expectation of an increased rate of error reduction. A substantial challenge to the proposed account would be a study modeling ExPERRs that found this measure not to be predictive of the valence or intensity of metacognitive feelings.

Within the proposed PP account of all metacognitive feelings, future work could develop computational models of each metacognitive feeling and apply them to behavior in experimental settings. We have already reviewed steps in this direction when discussing germane models of individual metacognitive feelings, such as the conception of the aha experience as the result of Bayesian reduction (Laukkonen et al., 2023), the feeling of pastness as conflicting sources of fluency (Brouillet et al., 2023b), surprise as the temporal interplay between perceptual input and the continuous fine-tuning of expectations (Gerten & Topolinski, 2019), or suspense as the expectation of upcoming valuable information (Li et al., 2021), which we interpreted as a feeling that models a sharp and uncertain change in the ExPERR. In our view, each metacognitive feeling would correspond to a model of prediction dynamics, their inferred causes, and specific changes to action policies. Therefore, researchers should model each metacognitive feeling separately while using the conceptual toolbox outlined in the current article. Such an approach is in line with what has recently been termed computational phenomenology, the application of methods from computational modeling to provide a formal model of the descriptions of lived experience (Ramstead et al., 2022). One advantage of PP is that its toolbox encompasses metacognition, interoception, and mental action. Of course, one might wonder whether the account could just model everything and whether PP has perhaps too many degrees of freedom. In the end, the success of PP for modeling metacognitive feelings will depend on the overall success of the different models of separate metacognitive feelings and how good those models are at explaining experimental evidence.

Another avenue of inquiry worth exploring concerns the neurobiological implications of the proposed account. One option is to manipulate neuromodulators such as dopamine and observe how they affect the relationship between metacognitive feelings and their associated mental action (for a suitable experimental design, see Clos et al., 2019). Existing neuroscientific evidence shows that prefrontal cortical subregions interact with interoceptive cortices (insula and cingulate) to support metacognition (for a review, see Fleming & Dolan, 2012). This neural mapping coincides with recent work within PP that suggests that interoceptive and exteroceptive predictions converge in the anterior insular cortex and that the cingulate uses neuromodulatory gain to control the precision of inferred interoceptive states, whereas regions of the prefrontal cortex are involved in contextualizing the inferences of these two interoceptive cortices over longer timescales (Allen et al., 2022). Further research could use the current account to explore this potential overlap and clarify the neural underpinnings of metacognitive feelings. Specifically, future work could explicitly manipulate the volatility of interoceptive changes by altering the underlying probability of an arousal change point (Behrens et al., 2007; Summerfield et al., 2011) and explore the resulting changes in metacognitive feelings. Such an endeavor could be helped by recent developments in the interoceptive technologies that encompass the direct manipulation of interoceptive signals, interoceptive illusions elicited by contextual cues, and emotional-augmentation technologies (for a review of these methodological developments within the PP framework, see Schoeller et al., 2022).

## Conclusion

In this article we advanced a PP account of how metacognitive feelings emerge and how they guide behavior. According to the proposed model, a wider system of active interoceptive inference serves to evaluate and regulate cognitive processes. The properties of the current stimulus, extant PE, and somatic and visceral signals all serve as cues for the ensuing rate of error reduction related to the ongoing cognitive activity. This predicted rate of error reduction corresponds to the phenomenal valence experienced by the subject. When a subject engages in a cognitive task that results in important expected changes in rate, metacognitive feelings arise to monitor and control the situation. In the current account, metacognitive feelings are understood not only as passive categorizations of a given state of affairs but also as active models that comprise both descriptive and directive dimensions. Their directive dimensions are expressed in the form of action policies that, through the adjustment of precision estimations, result in visceral changes in overt behavior and in the transformation of ongoing cognitive processes.

## Transparency

*Action Editor:* Rogier Kievit

*Editor:* Interim Editorial Panel

*Author Contributions*

P. F. Velasco and S. Loev contributed equally to this article and approved the final manuscript for submission.
